# Suvorexant for alcohol use disorder and post-traumatic stress disorder: study protocol for a phase II randomized clinical trial

**DOI:** 10.1186/s13063-026-09489-7

**Published:** 2026-02-03

**Authors:** Lara A. Ray, Steven J. Nieto, Karen Miotto, Larissa Mooney, Richard LeBeau, Jin H. Yoon, Joy M. Schmitz, Ron Acierno, Nathaniel W. Bailey, Jessica Jenkins, Jessica Vincent, Tracy Nolen, Shawn Hirsch, Alexis Williams, Scott D. Lane

**Affiliations:** 1 Department of Psychology, University of California at Los Angeles, 1285 Franz Hall, Box 951563, Los Angeles, CA 90095-1563 USA; 2 Department of Psychiatry and Biobehavioral Sciences, University of California at Los Angeles, Los Angeles, CA USA; 3 Center for Neurobehavioral Research on Addictions, University of Texas Health Science Center—Houston, Houston, TX USA; 4 Global Scientific Affairs, Merck & Co., Inc., Rahway, NJ USA; 5 Social, Statistical, & Environmental Sciences, RTI International, Research Triangle Park, Durham, NC USA

**Keywords:** Alcohol use disorder, Post-traumatic stress disorder, Insomnia, Suvorexant, Medication development

## Abstract

**Background:**

Alcohol use disorder (AUD) represents a highly prevalent, costly, and often untreated condition in the United States. Post-traumatic stress disorder (PTSD) represents a common comorbidity with AUD which worsens outcomes and decreases functional outcomes. Suvorexant (SUV) shows clear promise as a novel therapeutic candidate to treat AUD and PTSD.

**Methods:**

This study features a promising compound (i.e., suvorexant), the application of a well-established human laboratory paradigm (i.e., alcohol cue reactivity), and a novel early efficacy laboratory model (i.e., practice quit attempt) to provide a cost/time-efficient evaluation of safety and initial efficacy of suvorexant for AUD with comorbid PTSD. Additionally, by collecting both objective and subjective sleep measures, the study provides an assessment of a putative mechanism through which suvorexant jointly addresses an intervening variable common to both AUD and PTSD.

**Discussion:**

The combination of human laboratory modeling and real-world clinical outcomes provides a unique and synergistic set of data that can advance the development of suvorexant and identify its behavioral mechanisms of action. The recruitment of individuals with AUD and PTSD with sleep disturbances and who are intrinsically motivated to quit is a novel approach to screening pharmacotherapies by bridging the gap between experimental studies with non-treatment seekers and clinical trials with treatment-seeking individuals.

**Trial registration:**

ClinicalTrials.gov NCT06679062 “Suvorexant for Treatment of AUD and PTSD (SUV).” Registered on November 12, 2024.

## Administrative information

**Table:** 

Title {1}	Suvorexant for alcohol use disorder and post-traumatic stress disorder: study protocol for a phase II randomized clinical trial
Trial registration {2a and 2b}	ClinicalTrials.gov; NCT06679062
Protocol version {3}	Version 1; dated 03/2024
Funding {4}	Department of Defense (DOD), Congressionally Directed Medical Research Programs, Pharmacotherapies for Alcohol and Substance Use Disorders Alliance (PASA)
Author details {5a}	Department of Psychology, University of California, Los Angeles, Los Angeles, CA (LAR, SJN, RL, JJ) Department of Psychiatry and Biobehavioral Sciences, University of California, Los Angeles, Los Angeles, CA (LAR, KM, LM) Faillace Department of Psychiatry and Behavioral Sciences, UT Health Houston, Houston, TX (JHY, JMS, RA, JV, SDL) Merck & Co., Inc., Rahway, NJ, USA (NWB) Social, Statistical, & Environmental Sciences, RTI International, Research Triangle Park, NC (TN, SH, AW)
Name and contact information for the trial sponsor {5b}	Pharmacotherapies for Alcohol and Substance Use Disorders Alliance (PASA) pasa@rti.org
Role of sponsor {5c}	The sponsor is responsible for the study design and will play a part in the collection, management, analysis, and interpretation of data; writing of the report; and the decision to submit the report for publication

### Introduction

#### Background and rationale {6a}

Alcohol use disorder (AUD) represents a highly prevalent, costly, and often untreated condition in the United States (US). Post-traumatic stress disorder (PTSD) represents a common comorbidity with AUD which worsens outcomes and decreases functional outcomes. Pharmacotherapy offers a promising avenue for treating AUD and PTSD and for improving clinical outcomes for this debilitating comorbidity [1]. While there are FDA-approved medications for AUD and PTSD, these medications are only modestly effective. Therefore, the development and validation of novel treatment targets and compounds is a high-priority research area [2].

Suvorexant (SUV) (Merck & Co., Inc., Rahway, NJ, USA) was the first dual orexin receptor antagonist (DORA) approved for the treatment of insomnia in the US in 2014. A post-marketing trial evaluated the treatment of insomnia with suvorexant in patients with mild to moderate Alzheimer’s disease, and the resulting positive data was included in a label update in 2020. The orexin/hypocretin system is well known for its role in sleep-wake regulation but has more recently been implicated in AUD [3]. Animal models demonstrate that orexin 1 receptor antagonists reduce alcohol drinking in alcohol-dependent mice and alcohol-preferring rats [4–8]. Orexin-2 antagonists also reduce alcohol drinking and reinstatement/relapse in mice and rats [9–11]. Similarly, dual orexin antagonists (DORA) reduce alcohol consumption in alcohol-preferring rats [12, 13]. Given the effectiveness of orexin antagonists in reducing alcohol drinking at the preclinical level of analysis, suvorexant has garnered interest as a drug that can be repurposed to treat AUD [14]. However, these findings have yet to be translated in humans. Understanding if suvorexant can improve drinking outcomes and alleviate PTSD symptoms in a real-world setting is fundamental to the development of SUV as an AUD and PTSD treatment. Moreover, it is critical to elucidate the behavioral mechanisms by which SUV acts.

In addition to the well-established effects in regulating sleep, 15+ years of preclinical data evidence, a key role of the orexin system and DORAs in attenuating (1) drug seeking and self-administration (including alcohol), and (2) PTSD-relevant symptoms including reductions in stress/anxiety [15, 16]. SUV has two key advantages over more traditional somnolent medications (e.g., benzodiazepines) in this population: (1) it uniquely re-regulates polysomnographic (PSG)-measured sleep architecture rather than simply improving onset latency and total sleep time, moving sleep patterns toward homeostasis; and (2) it does not potentiate central nervous system (CNS) depression with alcohol. A phase II clinical trial of SUV for AUD and comorbid insomnia (prevalent in AUD) launched in Australia in 2020 (NCT 03897062) [14]. The National Institute on Drug Abuse (NIDA) medications development branch has placed DORAs/orexin antagonists on its “10 most-wanted” list [17].

Insomnia, addiction, and the orexin system are strongly connected [5]. The orexin system comprises two receptors (ORX-1, ORX-2) and corresponding neuropeptides (ORX-A, ORX-B, respectively) with probable unique functions related to sleep (ORX-2), stress/anxiety modulation (ORX-1), and energy homeostasis [6, 7]. Note that each of these areas is highly problematic in PTSD, with sleep problems being the most cited comorbid complaint in veterans with PTSD [8] followed by stress/anxiety symptoms. Similarly, disruption of sleep and heightened stress-induced cue reactivity are also highly prominent in AUD, driving motivation for ongoing use and relapse after abstinence [9, 10]. DORAs, including the proposed Food and Drug Administration-approved medication suvorexant, modulate both ORX-1 and ORX-2, serving as a biologically ideal target for addressing the overlapping symptoms in AUD and PTSD. Specifically, DORAs can effectively regulate sleep and restore normal sleep architecture [15] and attenuate PTSD symptoms [16] and motivation to use alcohol in preclinical models. The University of Texas Health Science Center at Houston (UTHealth) group published the first human study of orexin antagonism in substance use disorder (cocaine) with demonstrated effects on sleep and HPA-axis reactivity [18].

SUV shows clear promise as a novel therapeutic candidate to treat AUD and PTSD. Animal studies have shown that orexin receptor antagonists reduce both alcohol administration and relapse-like behavior in alcohol-dependent mice and alcohol-preferring rats [4–13]. However, to date, no published studies have translated these findings to clinical AUD samples or AUD + PTSD comorbidity. Understanding if suvorexant can improve drinking outcomes and alleviate PTSD symptoms in a real-world setting is fundamental to the development of this dual orexin receptor antagonist as an AUD + PTSD treatment. Moreover, elucidating the behavioral mechanisms by which suvorexant acts is also critical. Specifically, animal research has shown multiple potential mechanisms underlying suvorexant efficacy including reducing craving, reducing motivation, and relieving withdrawal symptoms. The protocol team has intentionally set a “wide net” of potential mechanisms given the scarce literature on the effects of suvorexant in AUD. Consideration of sleep, craving, and mood as behavioral mechanisms is consistent with work by Mason et al. [19–21] in medication development.

This study features a promising compound (i.e., SUV), the application of a well-established human laboratory paradigm (i.e., alcohol cue reactivity), and a novel early efficacy laboratory model (i.e., practice quit attempt) to provide a cost/time-efficient evaluation of safety and initial efficacy of suvorexant for AUD with comorbid PTSD. Additionally, by collecting both objective and subjective sleep measures, the study provides an assessment of a putative mechanism through which suvorexant jointly addresses an intervening variable common to both AUD and PTSD. The combination of human laboratory modeling and real-world clinical outcomes provides a unique and synergistic set of data that can advance the development of suvorexant and identify its behavioral mechanisms of action. The recruitment of individuals with AUD + PTSD with sleep disturbances and who are intrinsically motivated to quit is a novel approach to screening pharmacotherapies by bridging the gap between experimental studies with non-treatment seekers and clinical trials with treatment-seeking individuals. This is relevant given the wide range of differences between treatment seekers and non-treatment seekers with AUD [22, 23]. The study will also rely on an in vivo cue-reactivity paradigm which is highly consistent with the Human Laboratory Program approach by the National Institute on Alcohol Abuse and Alcoholism, a gold standard in medication screening. Assessments of PTSD symptomatology and sleep provide a comprehensive picture of clinical mechanisms of action for this patient population. Lastly, the novel practice quit paradigm has high ecological validity and the potential to detect clinically meaningful effects. Together, these design features enhance the study innovation and its overall scientific impact.

The successful completion of this study will set the stage for a larger phase II clinical trial of suvorexant for AUD and comorbid PTSD. Given the high prevalence of AUD + PTSD and the high acceptability of SUV as a therapeutic agent, the study has the potential to be transformative in the treatment of AUD + PTSD and in uncovering the therapeutic benefits of SUV.

### Objectives {7}

The primary objective of this study is to assess the initial efficacy of SUV (20 mg) in improving sleep metrics.

The secondary objectives of this study are:

- To assess the effect of SUV (20 mg) on cue-induced alcohol craving.
- To evaluate the effect of SUV (20 mg) on the mean number of drinks per drinking day.
- To evaluate the effect of SUV (20 mg) on drinks per day during the medication period over a 14-day quit attempt.
- To assess the initial efficacy of SUV (20 mg) in reducing PTSD symptoms.

The exploratory objectives of this study are:

- To assess the efficacy of SUV (20 mg) in improving sleep latency onset (SLO), wake after sleep onset (WASO), and total sleep duration via actigraph data.
- To evaluate the effect of SUV (20 mg) on National Institutes of Health Patient-Reported Outcomes Measurement Information System (PROMIS) sleep scale scores.

### Trial design {8}

This is a randomized, double-masked, placebo-controlled study to evaluate preliminary efficacy and safety of SUV (20 mg) for sleep disturbance in AUD and co-occurring PTSD symptoms in approximately 76 randomized men and women veterans and non-veterans between the ages 21 and 65. Following a 7-day placebo run-in, participants will be randomly assigned to receive SUV (10 mg (days 0–6) and 20 mg (days 7–13)) or matched placebo. Participants will be stratified on site, sex, and level of sleep disturbance (Insomnia Severity Index (ISI) score). Post-randomization, all participants will complete an alcohol cue-reactivity paradigm prior to the initial dose of study medication. The alcohol cue-reactivity paradigm is an established laboratory assessment of craving during which participants are exposed to real alcohol and water cues in a bar laboratory setting. Participants will then take their first dose of medication. Participants will begin the real-world practice quit attempt, during which they will attempt to stop drinking for 2 weeks. Participants will complete daily virtual diaries and visits to assess sleep, past-day drinking, and alcohol craving. Participants will return to one of the clinical sites on study day 14 to complete an alcohol cue-reactivity session, to assess post-medication craving. PTSD symptoms will be assessed via Clinician-Administered PTSD Scale for Diagnostic and Statistical Manual of Mental Disorders, Fifth Edition (DSM-5) (CAPS-5) and PTSD Checklist for DSM-5 (PCL-5) at baseline, at day 7, and at day 14 of treatment with SUV or matched placebo.

## Methods: participants, interventions, and outcomes

### Study setting {9}

This study will utilize 2 sites; one in Houston, TX (UTHealth) and one in Los Angeles, CA (UCLA). The UTHealth’s primary recruitment center for veteran and non-veteran population will be the UTHealth Trauma and Recovery Center (TRC). UCLA Department of Psychology will be the primary research site in California and will collaborate with the West Los Angeles VA Medical Center to recruit the veteran population.

### Eligibility criteria {10}

To be enrolled in this study, participants *must meet* the following criteria.

1. Age between 21 and 65.
2. Meet current (i.e., past 12 months at day −7/−6) DSM-5 diagnostic criteria for moderate or severe AUD as determined by the MINI.
3. Currently experiencing PTSD symptoms at screening (day −7/−6) as indicated by PCL-5 cut-score > 30.
4. Intrinsic motivation to reduce or quit drinking (defined as self-reported intention at screening to reduce or quit drinking within the next 6 months) and to receive PTSD treatment.
5. Must have an ISI score equal to or >7 (subthreshold insomnia). ISI score below 7 at screening will not be included or proceed beyond the screening day.
6. Agree to abstain from all other sleep medications (starting at day −7).
7. Have a place to live in the 2 weeks prior to randomization (day 0) and not be at risk that s/he will lose his/her housing in the next month.

To be enrolled in this study, participants *must not meet* the following criteria.

**Fig. 1:**
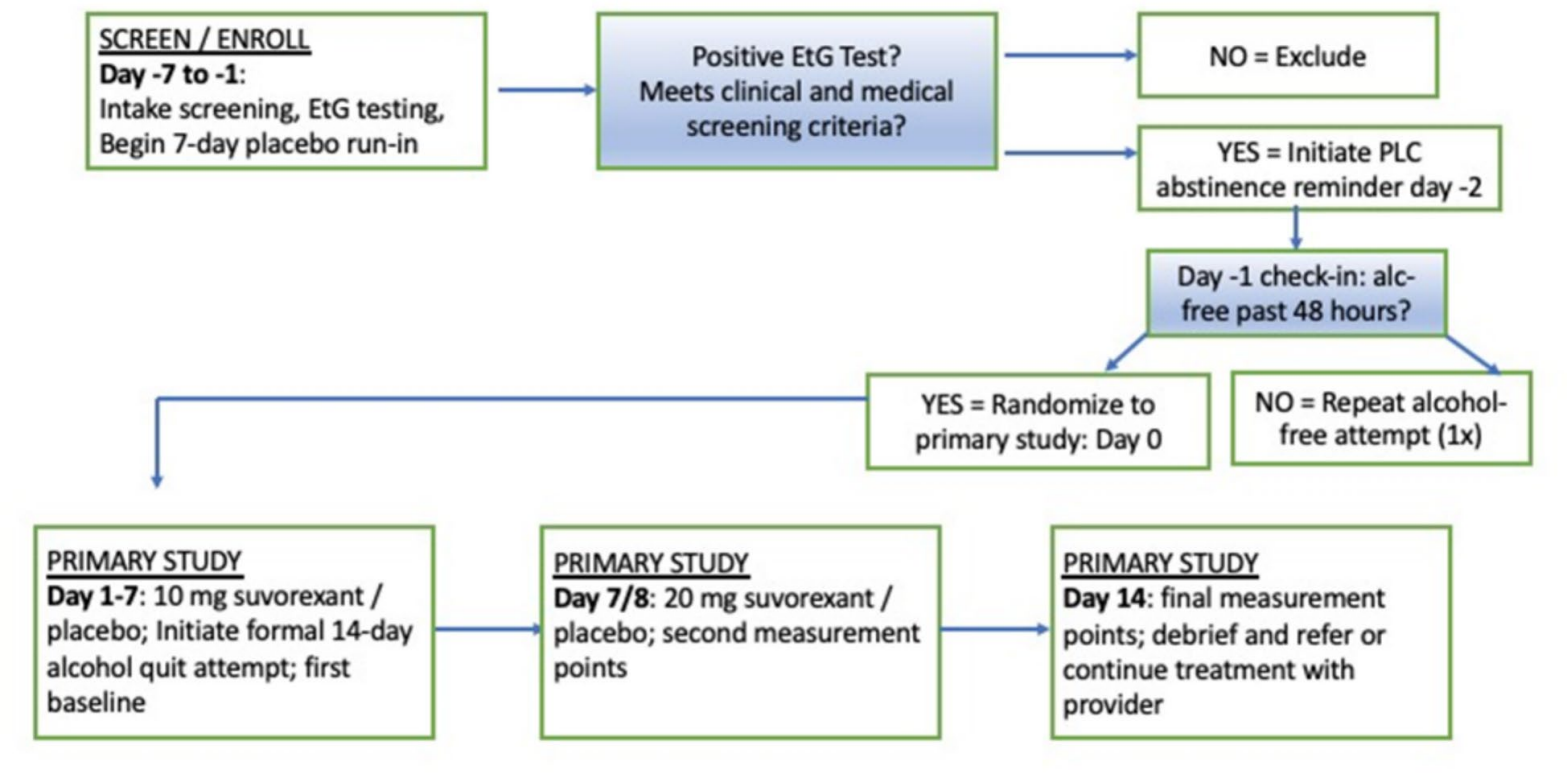
The study design is a double-blind, randomized, phase II study comparing the effectiveness of 20 mg oral suvorexant (SUV) versus placebo (PLC) (1:1), with a 1-week placebo run-in phase for all participants

1. A current (past 12 months at day −7/−6) DSM-5 diagnosis via the Mini-International Neuropsychiatric Interview (MINI) of substance use disorder for any substances other than alcohol, nicotine, or marijuana (< moderate level on DSM-5).
2. A lifetime DSM-5 diagnosis via the MINI of schizophrenia, bipolar disorder, or psychotic disorder.
3. Positive urine test for any recreational drugs other than marijuana at screening (day −7/−6).
4. Current clinically significant alcohol withdrawal (i.e., score ≥ 10 on the Clinical Institute Withdrawal Assessment Alcohol Scale Revised (CIWA-Ar)).
5. Currently pregnant, nursing, or no reliable method of birth control (females only).
6. Any clinically significant medical condition that would preclude safe participation in the study (e.g., narcolepsy, seizure disorder, or other clinically significant cardiovascular, hematologic, hepatic, renal, neurological, or endocrine disorders).
7. Use of suvorexant (within 30 days of day −7).
8. Currently on prescription medication that contraindicates use of suvorexant (including moderate or strong cytochrome P450 3 A modulators (CYP3A inhibitors and inducers)).
9. Hepatic insufficiency (AST/ALT > 5× upper limit of normal (ULN)).
10. Suicidal ideation determined by ≥ moderate Columbia Suicide Severity Rating Scale.
11. Inability to provide evidence of 48-h alcohol abstinence (self-report, breath alcohol (BrAC), ethylglucuronide (EtG) testing) at day 0 and failure after second attempt at 48-h abstinence (see Fig. 1).

### Who will take informed consent? {26a}

Potential participants are given ample time to consider the informed consent. They may have as much time as needed (no time limit) to read and consider the risks and benefits of the study participation, and they may involve family members, significant others, and the primary treatment team in the decision on whether or not to participate in the study.

Prior to entering the study, a member of the study team provides the potential participant with detailed information regarding the study’s purpose, procedures, potential risks and benefits, alternative treatment, compensation, and other required elements. This is done by a study team member in a private setting and documented on a signed and dated informed consent form. The investigators are responsible for answering questions pertaining to the study medication and their side effects. A participant’s willingness to take part in the study is subsequently documented in the medical records. If a participant agrees to participate, his/her consent is recorded on the Institutional Review Board (IRB)-approved informed consent form. Informed consent requires that the participant understands the details of the study, including its risks and benefits, and agrees without coercion to participation. The signed and dated consent form is distributed to participant’s study file (original), research pharmacy files (copy—if randomized), and participant (copy). A note is entered by the study staff into the participant’s medical record to document the process of informed consent.

All participants must have the capacity to provide their own informed consent. Surrogate consent is not allowed. Only those patients with capacity for informed consent are eligible. Persons who lack capacity to give independent informed consent (i.e., severe cognitive disorder such as dementia, severe traumatic brain injury, or other known cognitive or psychotic disorder that impairs decision-making) are not eligible to be enrolled. Persons with a terminal illness, minors, and prisoners should not be recruited or enrolled in this study.

Protection of participants from harm must be balanced against the potential for benefit to participants themselves, and to other persons with their disorders, that may arise from research participation. Since new treatments must eventually be tested in persons suffering from the condition, a policy totally excluding vulnerable participants from research would preclude the development of improved treatment for persons with AUD or PTSD. This study specifically recruits veterans, service members, first responders, and community volunteers with AUD or PTSD, which may be considered a vulnerable patient population. Vulnerable populations, i.e., prisoners, individuals with mental retardation, minors, persons with dementia or severe cognitive disorders, and persons deemed legally incompetent, are not eligible for this study. The decision-making capacity of individuals with AUD or PTSD at this stage of recovery is typically intact; however, veterans specifically are accustomed to taking and following direct orders, which requires a greater need for the researcher to prevent coercion, either directly or indirectly. Potential participants are given ample time to read and consider the informed consent, with other treatment options explained in the consent form. Family members, significant others, and primary treatment teams may also be involved in the decision-making process if the participant wishes. Veterans specifically are also informed that declining participation in a study does not change their eligibility for VA services, treatment, disability, or other VA benefits. Those individuals who are economically or educationally disadvantaged may be considered vulnerable. The VA population includes individuals in this category; however, the study keeps payments to participants at a minimum to avoid coercion based on economic hardship. In addition, the informed consent is written at a grade school level of education to minimize vulnerability to the educationally disadvantaged.

### Additional consent provisions for collection and use of participant data and biological specimens {26b}

Participant data and biological specimens used to evaluate eligibility and study compliance (i.e., urine sample, ECG, CBC, CMP) will be disposed of after testing.

## Interventions

### Explanation for the choice of comparators {6b}

The trial is placebo-controlled due to there being no universal standard-of-care medication for AUD or PTSD treatment.

### Intervention description {11a}

The study will utilize 10 mg and 20 mg SUV tablets and matching placebo obtained from Merck & Co., Inc., Rahway, NJ, USA. One SUV tablet (10 mg/20 mg) or placebo tablet will be taken orally once daily 30 min before bed with at least 7 h remaining prior to planned awakening. Each participant will be given a placebo run-in (day −7 to day −1). On day −2, participants will be prompted to initiate a 48-h abstinence period. If the study team determines appropriate quit attempts were successful on (verified by self-report + BrAC + EtG) baseline (day 0), the participant will be randomized to SUV or placebo and administered 10 mg SUV or placebo capsules for an additional 7 days (day 0 to day 6). On day 7, the participant previously randomized to SUV will be given 20 mg SUV, and the participant previously randomized to placebo will continue with placebo. The participant will be administered the remaining number of capsules (days 7–13).

### Criteria for discontinuing or modifying allocated interventions {11b}

Participants are free to discontinue study treatment or withdraw from participation in the study at any time, or the investigator may terminate a participant’s participation; however, these are two different actions that result in different study follow-up paths.

The investigator may discontinue study treatment for a participant or withdraw participation in the study if:

- Any clinical adverse event (AE), laboratory abnormality, or other medical condition or situation occurs such that continued receipt of study treatment or participation in the study would not be in the best interest of the participant.
- The participant meets an exclusion criterion (either newly developed or not previously recognized) that precludes further study participation.
- The participant demonstrates an inability to comply with the verbal and written study instructions and/or procedures or exhibits difficult behaviors (e.g., abusive, violent, aggressive).
- The investigator decides that continuing in the study would be harmful to the participant.
- The participant uses illegal drugs while in the study.
- The participant needs to take medications that are not allowed while in this study.
- The participant is unable to keep appointments or complete study procedures as instructed.
- The participant has a bad reaction to the study drug such that s/he can no longer continue to take them.
- The study is canceled by PASA/DOD (project sponsor), Merck & Co., Inc., Rahway, NJ, USA (MISP grantor), the responsible IRB(s), or via recommendation of the DSMB.
- The patient is suspected of abusing the medication by taking larger doses than prescribed.

### Strategies to improve adherence to interventions {11c}

Potential participants will be educated on study procedures during the consent process and screening visit to help with adherence and understanding of the study. Various study retention techniques will be used throughout the study such as reminder calls and reimbursement for study visits to keep participants engaged in the study.

The study will also implement nightly text-based medicine prompts and verification through the Cell-phone Assisted Remote Observation of Medication Adherence (CAROMA) check-in. Video will capture the patient self-administering the capsule, increasing verification of compliance at high rates [24].

### Relevant concomitant care permitted or prohibited during the trial {11d}

Concomitant medications will be reviewed and documented by the study physician(s) at screening and all follow-up visits. Potential concomitant medications to be aware of include co-administration of CNS depressants and drugs that increase blood levels.

Per study exclusionary criteria, participants may not be taking the following medications: moderate CYP3A inhibitors, strong CYP3A inhibitors, strong CYP3A inducers, digoxin, and narcolepsy medications.

### Provisions for post-trial care {30}

Post-trial care may continue after study participation if any adverse events remain. The study physician will contact the participant via phone to follow up and close out any adverse events. Participants who drop out of the trial due to adverse events do not fall into this category.

### Outcomes {12}

The primary outcome is change in Insomnia Severity Index (ISI) score, measured as change from baseline to day 14.

Secondary outcomes include change in alcohol craving score from baseline to days 7 and 14, percent of total days abstinent during the 14-day practice quit attempt, mean number of drinks per drinking day as measured by the Timeline Follow Back, and change in PTSD symptoms as measured by change from baseline to days 7 and 14 in the CAPS-5 and PCL-5 score(s).

Exploratory outcomes include actigraphy sleep metrics for sleep latency onset (SLO), wake after sleep onset (WASO), and total sleep duration, as well as the change in PROMIS Sleep Disturbance *T*-score from baseline to day 14.

### Participant timeline {13}

The overall study design is outlined in Fig. 1. Assessments administered at each visit are detailed in Table 1.

**Table 1:**
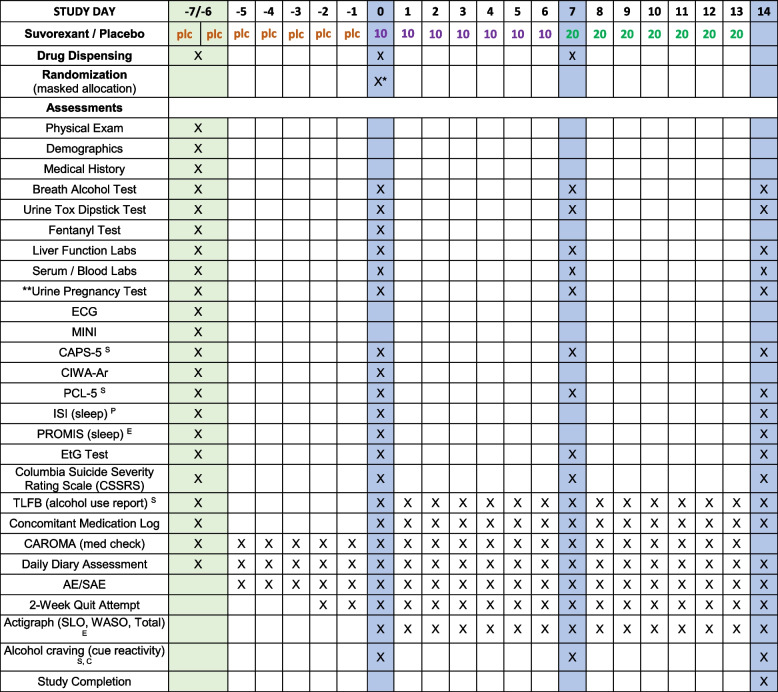
Procedures and measures administered at each study visit

*PLC* Placebo

* = Randomization will follow EtG confirmation of alcohol abstinence between Day –1 and Day 0

** = Urine Pregnancy Test is only required for female participants

P Primary Endpoint

S Secondary Endpoint

E Exploratory Endpoint

C Includes Alcohol Urge Questionnaire (AUQ), Profile of Mood States (POMS), and Visual Analog Scale (VAS)

Green Columns = The screening process may take up to two in-person days to complete

Blue Columns = Participants are to complete visit in person

#### Pre-screening, consent, and screening

Potential participants will initially be pre-screened by a trained research staff via phone. Participants meeting basic eligibility criteria will then be scheduled for a screening visit with a study staff member. The screening visit will take place in a private office where the research procedures, risks, and benefits associated with the study will be reviewed. Once a participant has signed the consent form, they will complete eligibility assessments across 1 or 2 days (days −7 and −6) that will be reviewed by trained research staff and the principal investigator (PI) to determine study eligibility.

#### Open label placebo lead-in

If determined eligible for the study, all participants will be given placebo for days −7 to −1 leading up to randomization (day 0) and reinformed that they are receiving placebo for this portion of the study. To ensure compliance with study drug self-administration, participants will be required to complete medication check-ins daily via CAROMA check-ins for the entire duration of their participation in the study.

On day −2, participants will be asked not to drink for the next 2 days, which will be confirmed by the EtG test on day 0. If the EtG test is positive, thus failing the participant, participants will be given an additional attempt to not drink for 2 days. If the EtG test is again positive, the participant will be screened out of the study. If the EtG test is negative during the first or second attempt, the participant will proceed to the rest of the baseline (day 0) assessments and randomization.

#### Baseline

On day 0, participants will be required to complete the visit in person. Participants will be considered enrolled in the study once they have provided consent, deemed to meet eligibility criteria, successfully completed the placebo lead-in period, and received confirmation of alcohol abstinence via the EtG test results. Participants will be randomized to suvorexant or matched placebo using a computer-generated algorithm coded in the electronic data capture (EDC) system with appropriate notifications to masked and unmasked study staff. Randomizations will be stratified on sex and level of sleep disturbance (ISI score) via the algorithm provided by PASA Data Coordinating Center (DCC).

At the conclusion of the visit, participants will be given an actigraph wearable device to capture daily sleep information. They are instructed to wear the device starting on day 0 through day 14. Participants will be administered either 10 mg SUV or matching placebo based on a masked randomization allocation for days 0 to 6.

#### Virtual follow-up

For days 1 to 6, participants will complete daily visits over the phone and complete online questionnaires on their past-days’ drinking, alcohol craving, mood, sleep, and PTSD symptoms via the EDC. Safety outcomes will also be collected through daily self-report. CAROMA check-ins will be completed daily at the time of medication self-administration.

#### In-person follow-up

On study day 7, participants will be scheduled for an in-person visit. At the conclusion of the visit, participants will be administered either 20 mg of SUV or matching placebo based on masked randomization allocation from baseline (day 0).

#### Virtual follow-up

For days 8 to 13, participants will continue the real-world practice quit attempt, during which they will attempt to stop drinking for the remaining 6 consecutive days. Participants will complete daily visits over the phone and complete online questionnaires on their past-days’ drinking, alcohol craving, mood, sleep, and PTSD symptoms via the EDC. Safety outcomes will also be collected through daily self-report. CAROMA check-ins will be completed daily at the time of medication self-administration.

#### Final study visit

On the final study day (day 14), participants will complete the last round of study assessments in person. Participants will return unused medication during this visit and cease CAROMA check-ins.

### Sample size {14}

Sample size for this study was determined based on the prior published studies of suvorexant effect on ISI which suggest that the standard deviation (SD) is between 6 and 8 points for the change from baseline. Based on a two-sided *t*-test of treatment effect with 80% power and alpha set at 0.05, the study would be able to detect a medium effect size (about 0.66, e.g., mean difference of 5.5 with SD of 7) with a total of 76 participants. This total number of participants includes those that complete screening, placebo lead-in, and at least day 0 study activities through randomization (and assuming no more than 7% attrition/loss to follow-up).

### Recruitment {15}

This study will utilize two sites: one in Houston, TX (UTHealth) and one in Los Angeles, CA (UCLA). The UTHealth’s primary recruitment center for veteran and non-veteran population will be the UTHealth Trauma and Recovery Center (TRC). UCLA Department of Psychiatry and Biobehavioral Sciences will be the primary research site in California and will collaborate with the West Los Angeles VA Medical Center to recruit the veteran population. Both sites will additionally utilize contracts with the BuildClinical social media platform to enhance enrollment.

## Assignment of interventions: allocation

### Sequence generation {16a}

Participants will be randomized in a 1:1 ratio to suvorexant or matched placebo using a covariate adjusted randomization algorithm, stratifying allocation by site, sex, and level of sleep disturbance via the ISI score. This algorithm is implemented within REDCap and is run once study coordinators ensure participant signs the informed consent, is eligible for the study, and enters all stratification variables.

### Concealment mechanism {16b}

Once a participant is randomized, the REDCap system will send an automatic email to each site’s pharmacy that includes the participant’s randomized treatment group. Each institution’s pharmacy will be responsible for labeling the unopened prefilled bottles according to the randomization information from the REDCap email. Unique labels for each masked dosing condition will be locked in the study pharmacy in a log/administration book and backed up on the respective university’s secure server.

### Implementation {16c}

The study pharmacist labels prefilled bottles of suvorexant and placebo with an appropriate KIT ID. Only the pharmacist knows which KIT IDs align with the active medication or placebo (the pharmacist will keep a log of this link file in a locked location). Once a participant is randomized, a research staff will pick up the appropriate KIT ID from the pharmacy and dispense it to the participant. The research coordinator will then fill out a Dispensing Case Report Form (CRF) documenting which KIT ID was provided to the participant.

## Assignment of interventions: blinding

### Who will be blinded {17a}

The study will be conducted in a double-blinded fashion in that both the participants and the site investigators and staff interacting with participants and assessing study outcomes will be blinded to treatment assignment.

### Procedure for unblinding if needed {17b}

The pharmacy will maintain the masking record which can be broken only upon request by the study investigators. If the blind is ever broken on any individual participant, the pharmacist will record that fact in the pharmacy dose record.

In instances where the study mask needs to be broken, permission and approval to break the mask will be obtained in advance from appropriate PASA Leadership if possible and does not impact the safety of the participant. Unblinded treatment assignment will come from the local site study pharmacist. Any breaking of the blind will be reported as a protocol deviation.

## Data collection and management

### Plans for assessment and collection of outcomes {18a}

Procedures and assessments specific to the study include medical, cognitive, and motor assessments, clinician-administered assessments, self-assessments, and alcohol cue reactivity.

#### Medical procedures and/or assessments

- Breathalyzer: Breathalyzer tests are used to measure participants’ breath alcohol concentration (BrAC). Samples >0.01 g/dl are considered positive. Results are kept in research records and not entered in medical records.
- Ethylglucuronide (EtG) testing: Ethylglucuronide is a byproduct of ethanol (alcohol that one drinks) and glucuronide, a common biological compound made in the liver that binds various toxins and drugs in the body that allows them to be excreted in the urine. When someone drinks even relatively small amounts of alcohol, EtG is formed and can be detected in the urine.
- Demographics: The interviewer asks the respondent for his/her date of birth, race, if he or she considers himself or herself to be Hispanic or Latino, and highest-grade level or degree received at the time of the interview.
- Medical history: This measure is a broad screening tool to collect information on a participants’ medical history, including recent hospitalizations, and check for conditions such as diabetes, liver disease, and kidney disease. It is comprised questions to cover the following areas: allergies, asthma, head, ears, eyes, nose, throat (HEENT), cardiovascular, renal, hepatic, pulmonary, gastrointestinal, musculoskeletal, neurologic, psychiatric, dermatologic, metabolic, hematologic, endocrine, genitourinary, reproductive system, seizure, infectious disease, inflammatory or auto-immune disorders, and diabetes. Screening will include questions to ensure patients are not currently taking any CPY3a inhibitors.
- Physical examination (PE): A targeted PE will include assessments of the head, eyes, ears, nose, throat, skin, thyroid, neurological system, lungs, cardiovascular system, abdomen (liver and spleen), lymph nodes, and extremities. Height and weight measurements will be taken during screening.
- Electrocardiogram (ECG): 12-lead ECGs will be obtained using a machine that automatically calculates heart rate and determines intervals for PR, QRS, QT/QTc, and PRT axes.
- Colombia Suicide Severity Rating Scale (C-SSRS): is a short questionnaire that screens for individuals at risk for suicide.
- Adverse events: All AEs occurring during the clinical trial will be collected, documented, and reported by the principal investigator/designated study staff according to the specific procedures detailed in section 22 below. Additionally, study staff will assess patients for any medical or psychiatric side effects by asking the participant “How have you been feeling since I saw you last?” and in a manner consistent with Systematic Assessment for Treatment of Emergent Events (SAFTEE) guidelines [25]. Study staff will also review the previous Adverse Event Form and inquire whether any of those events are continuing. Each new or unresolved adverse event will be recorded on the Adverse Event Case Report Form using a brief verbatim term, a severity ranking, and any additional description, according to the procedures. If an adverse event is reported that requires medical attention, it will be reported to a study clinician immediately for review. The PI/trained staff member will review each AE to assess their possible relationship to study medications and expectedness.
- Prior/concomitant medications (con-meds): Concomitant medications (con-meds) are other prescription medications, over the counter (OTC) drugs, or dietary supplements that a study participant takes in addition to the drug under investigation. Participants are asked to bring all their current medications with them at the time of their screening appointment.
- Actigraph: This wearable device will be used to measure the SLO and WASO variables in detecting activity and sleep patterns of participants. Participants will be given this device upon successful screening into the study.

#### Clinician-administered assessments

- Clinician-Administered PTSD Scale for DSM-5 (CAPS-5): Gold-standard in PTSD assessment. The CAPS-5 is a 30-item structured interview that can be used to make current (past month) diagnosis of PTSD, make lifetime diagnosis of PTSD, and assess PTSD symptoms over the past week [26–28].
- Clinical Institute Withdrawal Assessment Alcohol Scale Revised (CIWA-AR): This is a common instrument to assess and diagnose the severity of alcohol withdrawal, measuring 10 symptoms via a scale. This instrument takes about 2 min to complete and then scored by a medical professional. Cumulative scores of less than 8–10 indicate mild withdrawal. Next, scores of 8–15 indicate moderate withdrawal, and scores of 15 or more than 15 indicate severe withdrawal with impending possible delirium tremens.
- Mini-International Neuropsychiatric Interview for DSM-5: Assessment used to determine psychiatric diagnoses. This brief interview is structured and assesses DSM-5 current and lifetime psychiatric diagnoses. Note: Sections B, L, M, and MB will be omitted as these do not impact study inclusion/exclusion [29].
- Time-Line Follow-Back Assessment Method (TLFB): Interview technique that will be used to obtain quantity/frequency of alcohol consumption data for each day during the 30-day period prior to the study, throughout the period of study participation (measuring drinking on days outside of test session days) and the follow-up. Participants are given a blank calendar covering the time interval to be reconstructed and are asked to reconstruct retrospectively their drinking behavior over that interval. The process is facilitated by establishing anchor points (e.g., holidays, anniversaries, major national events). It can be scored to provide the number of days on which various levels of consumption occurred. The time-line method has good test-retest reliability and good validity for verifiable events. It has been used in numerous studies to compare pre- to post-treatment drinking [30].

#### Self-assessments

- PCL-5: The PCL-5 is a 20-item self-report measure that assesses DSM-5-based criteria for PTSD symptoms. Each item is rated on a 5-point Likert-type scale (0 = not at all; 4 = extremely) that indicates how much the participant has been bothered by an identified “worst” stressful event in the past month [31].
- Insomnia Severity Index Score (ISI): This is a brief questionnaire to screen for insomnia in participants. A scoring system is used to identify what degree insomnia (if any detected) is affecting daily life and sleep patterns.
- Patient-Reported Outcomes Measurement Information System (PROMIS): This system provides clinicians and study research staff measures of mental and physical health and overall well-being from a patient perspective.
- Daily diary assessment: Participants will complete daily diary assessments reporting on their mood, alcohol and cigarette craving, motivation to change, self-efficacy, pain, and drinking behavior from the previous day. Participants will complete the DDA electronically during the practice quit attempt (days 0–14). Research staff will distribute the link to the online daily diary assessment during each in-person and virtual visit.

#### Alcohol cue reactivity

This portion of the laboratory study consists of a presentation of a neutral cue (water) followed by an alcohol cue presentation and is used to assess alcohol craving. Immediately after each cue presentation, the participant will complete the Alcohol Urge Questionnaire (AUQ), Profile of Mood States (POMS), and Visual Analog Scale (VAS) based on the Alcohol Craving Questionnaire (ACQ).

- Neutral cue presentation: Individuals are instructed that they will be given a glass of water to handle for 3 min and that they may smell and handle the glass, but that they should not consume the water. In the presence of the research assistant, they are given the drink of water to hold and smell for 3 min. Following the presentation, the subject completes the assessments.
- Alcohol cue presentation: Individuals are instructed that they will be given their drink of choice to handle for 3 min and that they may smell and handle the drink, but that they should not consume the alcohol. In the presence of the research assistant, they are given their drink to hold and smell for 3 min. Following the presentation, the subject completes the assessments.

#### Clinical laboratory evaluations

- Urine ethylglucuronide (EtG) testing: This assay is used to detect whether participants have consumed alcohol in the last 42 to 78 h.
- Urine drug/toxicology screen: A Clinical Laboratory Improvement Amendments (CLIA)-waived multidrug cup is an immunochromatographic assay for rapid, qualitative detection of drug combinations and their principal metabolites in urine at specified cut-off concentrations. A multidrug cup will be used to test several drugs.
- Fentanyl screen: This dipstick test strip assay is used for rapid, qualitative detection of at least 10 fentanyl analogs, including carfentanil, in urine.
- Urine pregnancy test: This assay is used to detect whether female participants are currently pregnant.
- Liver function test: This assay is used to detect alanine transaminase (ALT) and aspartate transaminase (AST), alkaline phosphatase (ALP), gamma-glutamyl transferase (GGT), serum bilirubin, total protein, and albumin. These tests will signify affected areas of the liver where damage may be occurring or occurred and help with diagnosis and treatment options.
- Serum blood test: This assay will provide a broad overlook of the health of a participant. The standard battery of tests includes CBC with differential, chemistry panel, and hepatic panel (transaminases, albumen, LDH, cholesterol, etc.).

### Plans to promote participant retention and complete follow-up {18b}

Potential participants will be educated on study procedures during the consent process and screening visit to help with adherence and understanding of the study. Various study retention techniques will be used throughout the study such as reminder calls and reimbursement for study visits to keep participants engaged in the study. Compensation is as follows:

**Table:** 

Study activities	In-person visits + travel/parking	Virtual visits	CAROMA/Med compliance	Study completion
Payment	$75 (per visit)	$10 (per visit)	$5 (per day)	$100
Participants can earn up to $740, if they complete all study procedures				

### Data management {19}

The study will use Research Electronic Data Capture (REDCap) to store and database all electronic research data. Source documentation will be collected on hardcopy CRFs and entered into REDCap by research staff at the two clinical sites. Each site will have its own unique login credentials to REDCap, and each site will only be able to review its own study data. The REDCap database will be built and centrally monitored by the PASA DCC. The REDCap system goes through an internal User Acceptance Testing (UAT) phase to ensure it is bug-free and captures all appropriate study data prior to study launch.

Data cleaning in REDCap will be conducted on a regular basis to ensure the conduct of the trial follows the approved protocol, aligns with Good Clinical Practices (GCP) guidelines, and meets applicable regulatory requirements. Programmed system edit checks, manual edit checks, and ad hoc statistical reports will be generated to aid in the data cleaning process. The study audit trail is maintained within REDCap and captures all data changes made within the system including the date of the change, the original value, the new value, and the username of the individual making the change. Additionally, REDCap is built with branching logic and required fields to assigned variables to further increase data integrity.

All research staff entering data into REDCap are required to take a REDCap training course provided by the PASA DCC. All data management practices are outlined in the Data Management Plan (DMP).

### Confidentiality {27}

Study staff will maintain appropriate medical and research records for this study, in compliance with International Council for Harmonisation (ICH) E6 and regulatory requirements for the protection of confidentiality of participants. Physical research data will be stored in locked files in secure rooms at the UTHealth and UCLA sites, while electronic research data will be stored on locked computers on secure UTHealth and UCLA servers. Data will be kept for at least 7 years beyond the final publication date or submission to the FDA. De-identified data will be destroyed by shredding, and electronic data will be deleted from databases. Data and study files may be retained longer if required by other federal regulations or sponsor archive requirement.

Participant’s contact information will be securely stored at each clinical site for internal use during the study. Study participant research data will be transmitted to and stored at the PASA DCC. This will not include the participant’s contact or direct identifying information. Rather, individual participant research data will be identified by a unique study identification number. The REDCap system used by the study is secured, password-protected, and HIPAA-compliant.

To address concerns about confidentiality, the protocol team will obtain a Certificate of Confidentiality (CoC). CoCs allow researchers to refuse to disclose names or other identifying characteristics of research participants in response to legal demands.

### Plans for collection, laboratory evaluation, and storage of biological specimens for genetic or molecular analysis in this trial/future use {33}

Samples collected under this protocol will not be stored and will be analyzed immediately for participant health safety purposes and toxicology screens. Samples are destroyed after analysis.

## Statistical methods

### Statistical methods for primary and secondary outcomes {20a}

The study will test the single hypothesis that change in ISI score between baseline and day 14 differs between suvorexant and placebo at 14 days using a 0.05 significance level. We will also test treatment differences for five secondary measures: change in AUQ score, change in PCL-5 and CAPS-5 score, comparison of number of mean drinks per drinking day, and count of days with no alcohol consumption. Secondary analyses will be adjusted for multiple comparisons using the Benjamini-Hochberg false discovery rate correction to maintain the nominal alpha level of 0.05. All other statistical comparisons will be descriptive. All analyses will be based on the intention-to-treat population, and no other analysis populations are planned. All analysis and methods will be fully detailed in the study’s Statistical Analysis Plan (SAP).

For the primary outcome, comparison of the change in ISI score from baseline to day 14 between treatment groups will be analyzed using a linear model for continuous outcomes that accounts for treatment, site, sex, and baseline ISI score (see proposed model). Adjusted estimates of the change in ISI score between treatment and within treatment and corresponding 95% confidence intervals will be produced.

$$\mathrm{Y}_{\mathrm{i}} = \upbeta_{0} + \upbeta_{1}\mathrm{Treatment}_{\mathrm{i}} + \upbeta_{2}\mathrm{Site}_{\mathrm{i}} + \upbeta_{3}\mathrm{Sex}_{\mathrm{i}} + \upbeta_{4}\text{Baseline ISI} + \epsilon_{\mathrm{i}}$$

where *Y*_*i*_ is the change in ISI score from baseline to day 14, *β*_0_ is the intercept, *β*_1_ through *β*_4_ are coefficients, and *ε*_*i*_ is the error term which is assumed normally distributed.

A similar model will also be run for the secondary outcome of mean number of drinks per drinking day, while a Poisson model will be explored for the number and percent of days abstinent during the 13-day quit attempt period, accounting for treatment, site, sex, and baseline ISI score.

A mixed linear model for repeated measures will be used to estimate treatment differences for the change AUQ, PCL-5, and CAPS-5 scores from baseline to days 7 and 14. The model(s) will have fixed effects for treatment, time interval (as a categorical variable), treatment-by-time interaction, and the stratification effects of site, sex, and baseline ISI score. The baseline score for each outcome measure will also be included as a term in the model, as appropriate.

### Interim analyses {21b}

No formal interim analysis is planned; however, the study will be monitored by a Data Safety Monitoring Board (DSMB) committee. If the DSMB requests any sample size re-calculations or formal futility analyses, an addendum to the SAP will be made prior to implementation.

### Methods for additional analyses (e.g., subgroup analyses) {20b}

All primary and secondary outcomes will be explored for a sex by treatment interaction to determine if there is any signal of a differential treatment effect by sex. This interaction term will be included in models as a separate exploratory analysis goal. Since these analyses are exploratory in nature, no adjustment for multiple hypothesis testing will occur, and results will focus descriptively on point estimates and 95% confidence intervals (and opposed to *p* values to identify statistically significant differences).

### Methods in analysis to handle protocol non-adherence and any statistical methods to handle missing data {20c}

The intention-to-treat (ITT) population will be used for all planned analysis, with data from all participants analyzed according to the arm to which they were randomized irrespective of the amount of intervention received. This approach ignores noncompliance, protocol deviations, withdrawal, and lost-to-follow-ups. Analysis of the ITT population avoids overoptimistic estimates of the efficacy of an intervention resulting from the removal of non-compliers, accepting that protocol deviations occur in actual clinical practice.

Analysis of the secondary outcomes will be based on a mixed-effect model of repeated measures (MMRM) using data from all time points to account for missing data and thereby maximize information used for the analyses. These models treat missing data as ignorable missing, assuming any missing data are missing at random. No imputation processes will be used to replace missing data. However, every effort will be made to minimize missing data. Additionally, because missing observations have the potential to alter the results of analyses, we will examine whether the pattern of missing data is different among the groups.

### Plans to give access to the full protocol, participant-level data, and statistical code {31c}

After all data have been collected and the results of the study have been published, the full protocol will be provided as a supplement to the manuscript and/or uploaded to ClinicalTrials.gov. Additionally, the SAP will also be provided as a supplemental file to the manuscript. Although no formal statistical code will be provided due to proprietary concerns, the SAP will include general analysis code for each study outcome. Within the bounds of relevant IRB approvals, de-identified raw data from this study will be deposited in an appropriate, DOD-approved, publicly available data repository (i.e., National Institute of Mental Health Data Archive).

## Oversight and monitoring

### Composition of the coordinating center and trial steering committee {5d}

As mentioned earlier, the clinical study teams will collaborate with the PASA DCC to implement PASA-developed procedures for ensuring compliance with FDA requirements and to provide general oversight and monitoring. The PASA DCC is responsible for performing all data management and analysis tasks, performing initial, interim, and closeout site monitoring visits, and performing all DSMB administrative tasks. The clinical team will collaborate with PASA to implement:

1. Procedures to meet local IRB and United States Army Medical Research and Development Command Office of Human and Animal Research Oversight (OHRO) requirements for the conduct of clinical trials and the protection of human subjects.
2. Procedures to ensure monthly enrollment rates progress as planned to fully power the study.
3. Procedures to assist with the preparation of quarterly written progress reports, annual reports, and a final comprehensive report.
4. Procedures to develop, monitor, and close out the protocol, including submitting appropriate data and materials to allow for verification and review of protocol-related procedures.
5. PASA-developed procedures for the timely publication of major findings.

### Composition of the data monitoring committee, its role and reporting structure {21a}

The PASA DCC has established a DSMB to oversee this study. Members of the DSMB are independent of the study investigators and include representatives with substance abuse, pharmacology, and psychology/psychiatry expertise, a biostatistician, and an ethicist. Members will not have financial, scientific, or conflicts of interest which might interfere with their unbiased assessment of the progress of the trial. The DSMB will meet at least once every 4 months as specified in the PASA DSMB charter to review the study. The DSMB will monitor study progress and will have the ability to recommend that the trial be stopped for safety or futility.

The DSMB will provide recommendations to the DOD Grants Officer Representative (GOR) for further distribution to the Consortium Steering Committee and to the study PIs and sites for IRB submission. Recommendations will also be reported to the OHRO via annual reports for the study. While study halting rules will be considered by the DSMB in their deliberations about study safety and futility, final stopping recommendation criteria will be determined by the PIs/study team.

### Adverse event reporting and harms {22}

All AEs will be recorded on the study CRFs and entered into REDCap. Timely mandatory reporting is required for AEs that are serious, unexpected, and definitely, probably, or possibly related to the study drug. For all other events, the site will follow institutional policy for reporting to the IRB. Reporting to the DSMB will occur via study reviews occurring on a prescheduled frequency.

Each AE will be classified by the PI as serious or non-serious. Serious AEs (SAEs) will be reported by the site to their local IRB and to PASA within 24 h of identification. Initial reports will include as much information as possible but, at a minimum, should include the event term, onset date, severity, serious criterion, relationship to study drug, date of resolution (or continuing, if known), action taken with study drug because of the reported event, and an event outcome (if known). Initial reporting will be followed within 2 days by reporting missing AE characteristics and a description of the event.

Determination of whether the event may be unexpected and at least possibly related to the study drug will be made by the study clinical team in collaboration with PASA medical monitor. Any SAEs that are deemed related and unexpected will be submitted by PASA Leadership in conjunction with the study team in a safety report to the FDA, drug manufacturer, DSMB, and participating investigators. Clinical sites will follow local IRB guidelines for submission of any unexpected and related SAEs that occur. Participating investigators and DSMB will be notified by PASA Leadership in conjunction with the study team as soon as possible, but no later than 15 calendar days after it is determined the SAE qualifies for reporting. Furthermore, participating investigators and DSMB will be notified of any unexpected fatal or life-threatening suspected adverse reaction as soon as possible, but no later than 7 calendar days after initial receipt of the information.

### Frequency and plans for auditing trial conduct {23}

On-site or remote monitoring visits will be conducted by the PASA DCC separately at each clinic. The PASA DCC will conduct three separate visits at each site: initial, interim, and closeout visit(s). In addition to the PASA DCC’s central monitoring activities and DSMB meetings, the site monitoring visits serve as the methods used to control the quality of the study and document how the study plans to address potential study risks. The details and timing of each monitoring visit are documented in the Site Monitoring Plan (SMP).

### Plans for communicating important protocol amendments to relevant parties (e.g., trial participants, ethical committees) {25}

The PI will promptly inform PASA of any changes in recruitment or in the protocol that are relevant to safety, as well as any actions taken by the IRB as a result of its continuing review of the study. All necessary protocol changes will be submitted in writing as protocol amendments to the IRB by the PIs for approval prior to implementation.

### Dissemination plans {31a}

Trial results will be communicated primarily via publication. Merck & Co., Inc., Rahway, NJ, USA, will review the manuscript prior to submission for publication but will have no influence on results or interpretation.

## Discussion

Suvorexant (SUV) is a promising treatment for alcohol use disorder (AUD) and post-traumatic stress disorder (PTSD) due to the mechanism of action. Dual orexin receptor antagonism has demonstrated efficacy in restoring key sleep parameters in individuals with insomnia. Additionally, SUV has shown potential in preclinical studies to reduce alcohol self-administration and attenuate stress- and anxiety-like behaviors. The planned study will investigate the efficacy of SUV in improving sleep metrics, reducing alcohol craving, and alleviating PTSD symptoms in individuals with comorbid AUD and PTSD.

As of March 2025, and per ClinicalTrials.gov, the current study will be one of the first large-scale randomized clinical trials testing SUV in individuals with combined AUD and PTSD. This is a two-site trial, double-blind versus placebo with 1:1 randomization. Collection of laboratory-based alcohol-induced craving measures and PSG-validated actigraphy-based measures of sleep represents an innovative aspect of the study design, which seeks to elucidate mechanisms underlying the treatment effects without compromising the clinical trial design required to address the chief aims of sleep improvement and reduced drinking during a 14-day quit attempt. The potential of this trial to provide both initial efficacy signals and mechanistic insights for orexin-based AUD and PTSD pharmacotherapy stands to provide a justification for larger and longer multisite trials in this clinical population.

While this trial is fully powered to detect a significant effect for the primary aim (sleep as measured by the ISI), one limitation to be noted is that the other outcome measures (alcohol use over 14 days; cue-induced alcohol craving, PTSD symptoms) should be considered effect-size generating rather than confirmatory, and thus provisional in the context of the study. A second limitation is the 14-day trial duration. This duration was selected specifically to test a Go-signal for initial efficacy during a 14-day quit attempt period, similar to approaches used in previous AUD pharmacotherapy studies (e.g. [32],). This 14-day trial period can clearly only provide a provisional test of SUV on the selected outcomes. A third limitation is that the study funding period and protocol do not allow for the collection of follow-up data, e.g., 3- and 6-month outcomes. These above-noted limitations will necessitate larger multisite RCTs with more standard durations (i.e., 12–16 weeks) and appropriate long-term follow-up observations to advance SUV toward regulatory approval for the treatment of AUD and PTSD.

The successful completion of the current study will advance SUV, a promising novel compound with existing preclinical, safety, and efficacy data in SUD [18, 33] and PTSD [34]. A determination of statistically significant results for the primary and/or secondary aims is expected to provide justification for a larger, longer-duration confirmatory multisite trial in the treatment of co-occurring AUD and PTSD.

## Trial status

The current protocol version number is 1.0 (dated 03/2024). Recruitment is expected to begin in May 2025. Recruitment is expected to be completed in December 2025. Protocol modifications will be shared with appropriate parties (e.g., trial participants) and published on ClinicalTrials.gov. The results of this trial will be submitted for publication in a peer-reviewed scientific journal.

## Data Availability

PASA will ensure study data are deposited for data sharing with other researchers. Within the bounds of relevant IRB approvals and guidelines for protection of personally identifiable data, de-identified data from this study will be deposited in an appropriate, publicly available data repository (i.e., National Institute of Mental Health Data Archive).

## Abbreviations

AE: Adverse event
ALC: Alcohol
AUD: Alcohol use disorder
BrAC: Breath alcohol concentration
CAPS-5: Clinician-Administered PTSD Scale for DSM-5
CFR: Code of Federal Regulations
CIWA-Ar: Clinical Institute Withdrawal Assessment Alcohol Scale Revised
CLIA: Clinical Laboratory Improvement Amendments
CNRA: Center for Neurobehavioral Research on Addictions
CNS: Central nervous system
CoC: Certificate of Confidentiality
DCC: Data Coordinating Center
DEA: Drug Enforcement Agency
DoD: Department of Defense
DORA: Dual orexin receptor antagonist
DSM-5: Diagnostic and Statistical Manual of Mental Disorders, Fifth Edition
DSMB: Data and Safety Monitoring Board
ECG: Electrocardiogram
EDC: Electronic data capture
FDA: Food and Drug Administration
GCP: Good Clinical Practice
GOR: Grants Officer Representative
IND: Investigational new drug (application)
IRB: Institutional Review Board
ISF: Investigator site file
ISI: Insomnia Severity Index
NIAAA: National Institute on Alcohol Abuse and Alcoholism
NIDA: National Institute on Drug Abuse
mg: Milligrams
MISP: Merck Investigator Studies Program
MOP: Manual of procedures
OHRO: Office of Human Research Oversight
ORX: Orexin
OTC: Over-the-counter
PASA: Pharmacotherapies for Alcohol and Substance Use Disorders Alliance
PE: Physical exam
PCL-5: Post-traumatic Stress Disorder Checklist for DSM-5
PLC: Placebo
PROMIS: NIH Patient-Reported Outcomes Measurement Information System
PSG: Polysomnographic
PSQI: Pittsburg Sleep Quality Index Questionnaire
PTSD: Post-traumatic stress disorder
QC: Quality control
RCT: Randomized control trial
SAE: Serious adverse events
SAFTEE: Systemic Assessment for Treatment Emergent Effects
SLO: Sleep latency onset
SUD: Substance use disorder
SUV: Suvorexant
TLFB: Timeline Follow Back
TRC: Trauma and Recovery Center
UCLA: University of California Los Angeles
ULN: Upper limit of normal
UTHealth: University of Texas Health Science Center—Houston
WASO: Wake after sleep onset
